# The New Outlook in Oto-Laryngology
*The Watson-Williams Memorial Lecture, given at Bristol University on 8th March, 1955.


**Published:** 1956-04

**Authors:** R. Scott Stevenson

**Affiliations:** Consultant Ear, Nose and Throat Surgeon, Colonial Hospital, Gibraltar; Consulting Surgeon, Metropolitan Ear, Nose and Throat Hospital, London


### THE NEW OUTLOOK IN OTO-LARYNGOLOGY*

BY

R. SCOTT STEVENSON, M.D., F.R.C.S.Ed.

*Consultant Ear, Nose and Throat Surgeon, Colonial Hospital, Gibraltar; Consulting Surgeon, Metropolitan Ear, Nose and Throat Hospital, London*

### INTRODUCTION

My first duty, and indeed pleasure, is to thank the Council and Senate of the University of Bristol for the honour they have done me in inviting me to give the third Watson-Williams Memorial Lecture—a compliment I appreciate all the more when I have learned that my two predecessors were my distinguished colleagues, Mr. Mollison and Mr. Negus, both of whom I hold in the highest regard. I knew the late Dr. Patrick Watson-Williams over a period of some fifteen or sixteen years—and he was a man who in our specialty was regarded with affection as well as admiration. The *History of Oto-laryngology* refers to him in the words, " He is remembered as a genial and wise rhinologist." Patrick Watson-Williams will always be remembered in the history of medicine as a pioneer in Britain of the systematic study of the nasal sinuses, who emphasized in particular the connexion between chronic nasal sinus infection and general diseases. He qualified here as a doctor in the eighties and, like most of his contemporaries, graduated into laryngology by way of general medicine, for he was assistant physician to Bristol Royal Infirmary for seventeen years, until he became a full physician and was put in charge of the nose and throat department which, due mainly to his own efforts, was instituted in 1906; four years later there was added the ear department, which, as was usual then, had been in the charge of a general surgeon. As early as 1894 he had written a textbook on *Diseases of the Upper Respiratory Tract*. Dr. Watson-Williams was one of the standard-bearers who, along with Felix Semon, Peter McBride, Herbert Tilley and others, fought for the recognition of laryngology as a distinct specialty, using the word in its widest sense as including diseases of the nose as well as of the throat; he became a skilful surgeon, who devised new nasal operations and ingenious techniques, and he contributed copiously to the professional literature of his time, notably his book on *Chronic Nasal Sinusitis and its Relation to General Medicine*. It is the emphasis that he laid upon this important link that forms the permanent contribution of Patrick Watson-Williams to the art and science of medicine.

### THE " NEW OUTLOOK "

What is the " new outlook " in oto-laryngology that I have taken as my subject tonight? Briefly, it is the change of direction away from anatomy and pathology towards physiology and restoration of function, including rehabilitation. Today an ear, nose and throat specialist must be a psychologist as well as a craftsman, a physician as well as a surgeon. But do not let me be misunderstood: no doctor can become a competent oto-laryngologist without a sound general surgical training and a thorough grounding in anatomy and in pathology, testified to by the searching examinations that he must pass for a surgical fellowship. It is a pity, indeed, that life is so short; for the modern

* The Watson-Williams Memorial Lecture, given at Bristol University on 8th March, 1955.

oto-laryngologist, with his National Service, his general and specialized training and his examinations, is unlikely to get out into the world to earn his livelihood as a consultant before the age of 32 or 33.

### IMPROVED ANAESTHESIA

In my time, the outstanding advances in oto-laryngology have been due to the improved methods of anaesthesia, the discovery of the antibiotics, and the improved techniques in radiotherapy. Blood transfusion—which I remember as exciting and tentative in France in the 1914–18 War—might also be included, as improved methods of testing the patient, preparing and storing the blood, and carrying out the technique of transfusion have made it an everyday affair in surgery, and we use it sometimes in laryngological operations; on a few occasions I have even had to employ it in haemorrhage after tonsillectomy. But, when I look back, improved anaesthesia and the antibiotics are what have mainly changed our outlook in oto-laryngology.

In my younger days I had the distress of witnessing two deaths upon the operating table: in both, death was due to shock, and the cause was not too deep but too shallow an anaesthetic—one was chloroform, the other ether. But I can truthfully say that in the last twenty-five years I have never even had to think about the anaesthetic at an operation, and modern anaesthetic apparatus with new anaesthetic agents, intubation, intravenous barbiturates, relaxants and hypotensive drugs to lessen haemorrhage, have all made operations safer and more comfortable for both patient and surgeon. It was the work of the anaesthetists Magill and Rowbotham, working in plastic surgery units immediately after the 1914–18 War, that marked the beginning of modern anaesthetic technique. Most of the advances have been British in origin, and undoubtedly the improved training and improved status for the anaesthetist in this country have contributed greatly to these advances: the anaesthetist has become the " clinical physiologist " of the surgical team.

### ANTIBIOTICS

Nothing, however, in my time has altered diagnosis, treatment and prognosis in oto-laryngology so much as the introduction of the antibiotics. It is right that Sir Alexander Fleming has been universally honoured as the discoverer of penicillin, though he never developed it so that it could be applied clinically. But Fleming lit the spark, as someone will doubtless light the spark in cancer research which, despite the devotion of scientifically-minded workers all over the world, has somehow never found the right direction. He put his observations on record, in the *British Journal of Experimental Pathology* in 1929 and there it was for Florey and Chain and their colleagues at Oxford to find, in their wartime researches eleven years later.

I remember, when I was a First Assistant (what is now called a Registrar) in the Ear, Nose and Throat Department of University College Hospital some twenty-five years ago, and doing all the emergency operations, there was an influenza epidemic which especially attacked the ears, and I performed forty-two acute mastoid operations in five weeks; in 1953, my last year in London, I performed only three. Such is the enormous change in the incidence of acute mastoiditis. The general practitioner now gives adequate doses of penicillin in cases of acute otitis media and the infection seldom reaches the mastoid.

The old signs of mastoiditis are no longer seen—the tender swelling behind the ear, the displaced auricle, the profuse ear discharge, the tender and sagging bony meatus, the persistent rise of temperature and increased pulse rate. In spite of what I have just said about the comparative rarity of acute mastoiditis, I happen to have been asked to see two cases within the past six weeks.

One was a young soldier with an ear that had been profusely discharging off and on for a month and, as he had had a rise of temperature, had had full doses of penicillin, which were still being continued. When I saw him, there was no mastoid tenderness, no fever, a quiet pulse. But his meatus filled again with pus when I mopped it out, tuning-fork tests showed marked middle-ear deafness, and x-ray films showed cloudy mastoid cells on the affected side. A cortical mastoid operation revealed all his mastoid cells on that side full of glairy muco-pus.

The second case was a man of 30, who looked very ill when I saw him, with severe headache and some head retraction. There was no mastoid tenderness, but his doctor assured me that there had been mastoid tenderness a few days previously. His ear drumhead was intact and moved freely with Siegle's speculum, but his doctor assured me again that there had been a perforation a week or two before, and considerable ear discharge then. Examination showed some degree of middle-ear deafness. His temperature and pulse-rate were both normal. He had had several millions of units of penicillin, also aureomycin and terramycin. He looked so ill that I did not have an x-ray taken, but had him up to the operating theatre within the hour, when the second stroke of my gouge released a stream of foul-smelling pus from the tip cells of his mastoid—the micro-organisms were subsequently found to be penicillin-resistant. He was sitting up for lunch next day.

I give details of these two cases to show how greatly the signs of acute mastoiditis have changed, and how one must rely nowadays mainly on an accurate history, evidence of middle-ear deafness, and x-ray films of both mastoids—as well as, to be true, our old friend " clinical instinct ".

It is an interesting fact that in most hospital otological departments, as the number of simple mastoid operations for acute mastoiditis has gone down enormously, so the number of radical mastoid operations is going up. This operation is being done mainly for the removal of that curious and potentially dangerous result of chronic infection, cholesteatoma. Its diagnosis is said to be aided by x-ray examination of the mastoid but I have not found personally x-ray reports to be very accurate in this regard. There seems little doubt that the otologist nowadays knows his temporal bone in more accurate detail than he used to do, and this I attribute to his training for and performance of the fenestration operation under magnification. Whatever else may be said for or against the fenestration operation, at least it has taught the young otologist the minute anatomy of the temporal bone.

It was the discovery of the antibiotics that made the fenestration operation a practical proposition, for the early pioneers gave it up because of the danger of causing acute labyrinthitis and meningitis. Maurice Sourdille carried out his fenestration operation in stages in order to avoid these infections, but under a cover of penicillin the modern fenestration operation gives rise to no such fears.

I remember the late Albert Gray, who died in 1936, and who was the leading authority on the pathology of otosclerosis in his time, saying to me that he preferred a dead otosclerotic, whose labyrinth he could examine with his microscope, to a live otosclerotic, for whom he could do nothing. The fenestration operation does not cure the disease—the cause of which is still unknown—but it does (in the vast majority of cases) help the patient's deafness. In other words, it helps the physiology of the labyrinth without doing anything for its pathology.

Although much experimental work on vestibular function has been done, there still exists considerable uncertainty as to the precise functions of the various structures comprising the labyrinth. The fenestration operation, in which a new window is made in one of the bony semicircular canals in order that the sound-waves can by-pass the fixed stapes, presents an entirely new experiment on sound conduction in the internal ear. There is no doubt now, from a study of the results of the fenestration operation, that the entire labyrinth must be considered as a physiological unit; so long ago as

1934 the experimental work on the labyrinth of Ashcroft and Hallpike suggested that at least the saccule of the labyrinth might well be concerned in hearing, as well as the cochlea.

### THE NASAL SINUSES

With regard to the nasal sinuses—in which Dr. Watson-Williams was especially interested—the antibiotics have proved more useful in acute than in chronic sinusitis: in fact, my own experience has been that penicillin and the other antibiotics have been of no use in the treatment of chronic sinusitis, except that I give systemic injections of penicillin to help to avoid complications after operating on the nasal sinuses. I know that some of my rhinological colleagues use penicillin irrigations, sometimes with indwelling catheters, or a wax cream containing penicillin injected into the maxillary antrum, in the treatment of chronic sinusitis, but I am unconvinced that these measures are any more effective than irrigations of normal saline, unless operation proves to be necessary. I have always been inclined to be conservative in the treatment of acute nasal sinusitis, and in this troublesome condition penicillin and the newer antibiotics have proved a great blessing, employed, however, systemically, not locally—except that I have sometimes found penicillin aerosol helpful in early acute sinusitis.

I published a book on *Recent Advances in Oto-laryngology* in 1935 and a second edition in 1949, and it is very enlightening to compare the chapter on nasal sinusitis in the earlier edition with that fourteen years later. The earlier chapter was occupied mainly with the discussion of the technique of operative procedures upon the various nasal sinuses. In the later one the emphasis was much less on surgery and more on the rôle of nasal allergy and on the restoration of the function of the cilia of the nasal mucous membrane. So long ago as 1924 my old friend and colleague, Arthur Lowndes Yates, had shown that the ciliary movement is propagated along the surface of the nasal mucosa and maintains currents in the surface mucus which conduct microscopic bodies towards the ostium from within the sinus and towards the pharynx from within the nose. All operations on the nasal sinuses should be directed towards removing infection, bringing about free drainage, and restoring the activity of the cilia. Simple puncture and lavage of the maxillary antrum will often change the whole picture of a chronic nasal sinusitis, even a pan-sinusitis, and if an operation does have to be performed it should be radical; for example, it is many years since I performed an intranasal antrostomy—maxillary sinusitis will either clear up with repeated puncture and lavage with normal saline or will require the radical Caldwell-Luc operation.

We are rather inclined to use the word " allergy " as if we knew all about it, but in actual fact we still know very little about nasal allergy, except that it is a condition in which the tissues have become sensitized to certain agents (usually foreign proteins), leading to nasal irritation with sneezing, mucosal oedema, and thin watery nasal discharge containing eosinophil cells. I have come to the conclusion that nasal polypi are *always* due to nasal allergy and demand removal only for the sake of the nasal drainage; nowadays I am less inclined to undertake radical nasal operations to get rid of nasal polypi, and prefer to treat the allergy by anti-histamines and conservative treatment after simple removal of the polypi. Nasal drops or sprays may be helpful in these cases so long as they do not contain ephedrine, which is an irritant to the mucosa of nasal allergy.

### LARYNGEAL CANCER

In laryngology the antibiotics have made extensive, radical operations very much safer than they used to be, though improved methods of anaesthesia have also contributed to this end. I myself, as recently as 1946, wrote that " laryngectomy is an operation that even today many laryngologists hesitate to advise, especially since the introduction of radium and x-ray treatment". I was influenced then, I admit, by the recollection of the first four laryngectomies with which I was personally concerned many years previously, three of which proved fatal soon after the operation—one of them at his own hand. Looking back, I have to admit that my results in cancer of the larynx were not so good, in the years between the wars, as they should have been, owing to my reluctance to sacrifice the larynx: I was inclined to perform laryngo-fissure or hemi-laryngectomy, when today—under improved conditions—I would not hesitate to advise laryngectomy.

I remember well the attack that the late Mr. Lionel Colledge—who was a highly competent and experienced, if prejudiced, surgeon—made in 1945 upon the pretensions of radiotherapy in the treatment of cancer of the larynx. He said then that he believed that " the proportion of inoperable cases in radiological statistics had been exaggerated "; that " radiotherapy had failed to justify itself as a routine treatment for pharyngeal carcinoma, and still less for laryngeal carcinoma "; and that " one great difficulty in estimating the value of treatment either by x-rays or radium arises from the frequency with which the growth rapidly disappears and the organ regains a normal appearance—these cases are apt to be regarded as successful, but only too often within less than a year the tumour is found to be growing more rapidly than before and rapidly disseminating ".

But only ten years ago radiotherapy was looked upon, in the main, as a method merely of palliative treatment for inoperable cases of laryngeal cancer—out of 306 cases of cancer of the pharynx and larynx treated by radiotherapy by the late G. F. Stebbing before the end of 1936, only 21 were alive and free from symptoms three years later, but most of the growths were very advanced when first seen by him.

Today the attitude has changed: radiotherapy is, in my own experience and in that of others, the method to be preferred in cases of cancer of the larynx that are early and used to be considered " operable cases "; while surgery—usually laryngectomy—is on the contrary employed in those cases that are advanced and unsuitable for radiotherapy. Radiotherapy of malignant disease of the pharynx and of the nasal sinuses is unfortunately not in such a satisfactory state as in malignant disease of the larynx. Doctors everywhere have accepted the teaching of Chevalier Jackson and of St. Clair Thomson that no patient should be hoarse for a month without having his larynx examined by a laryngologist, but few of the cases of malignant disease of the pharynx or the nasal sinuses are diagnosed early enough. This is understandable in the pharynx, because of the slightness of the signs and symptoms: I have known a sarcoma of the pharynx incised more than once in mistake for a quinsy.

One-fifth of all patients with malignant disease of the nasal sinuses first consult a dentist and improvement in the results of the treatment of this refractory and fell disease cannot be anticipated until, at the slightest suspicion of malignancy, the nasal sinuses are x-rayed and a biopsy done. In my experience the best results so far have been brought about by a combination of surgery and radiotherapy. I remember the late Wilfred Trotter a good many years ago—before radiotherapy had reached its present excellence—telling me that surgery, with a good margin beyond the growth, would give hopeful results in many of these cases, for most of such growths, he thought, were not very malignant. The margin, however, may have to be somewhat extensive, and I have known a case in which the external carotid had to be tied, an eye and the floor of the orbit, half of the hard palate and the floor of the nose removed (by Mr. Alan Hunt, of the Royal Marsden Hospital). Perhaps I should add that with the aid of an efficient prosthesis this patient is able to earn his living at a catering job where he has to interview strangers, most of whom notice nothing unusual about his appearance.

The speciality of radiotherapy, it should be remembered, originated in Great Britain, and British radiotherapists—notably in Edinburgh, Glasgow, and Manchester, and at the Royal Marsden, the Middlesex and the Westminster Hospitals in London—still lead the world, as my friend Dr. Max Cutler, himself an outstanding radiotherapist of Chicago and now of Los Angeles, acknowledged to me only the other day.

The outlook in cases of laryngectomy has been completely transformed by the use of antibiotics and modern methods of anaesthesia, and one expects to see one's patient out of bed in twenty-four hours and out of hospital within ten days. We used to think the artificial larynx an important adjunct in helping a laryngectomized patient to speak—but today it has become obsolete. The patient who has had his larynx removed can be taught to speak without a great deal of difficulty: we still send such patients to the speech therapist for training before and after operation, but an experienced ward sister is a great help in this direction, and even more help is an old patient who has himself had a laryngectomy, looks well and speaks well enough, and so helps to give the patient confidence in himself. Mr. Tom Howie of Glasgow did a great deal for the laryngectomized patient when he founded the first Laryngectomy Club—an idea which has been copied at other centres, where old and new patients, who have lost their larynxes, regularly meet in a cheerful atmosphere of mutual assistance and teach each other how to swallow air and articulate with it.

### LARYNGEAL TUBERCULOSIS

In collaboration with Professor Heaf of Cardiff (then Medical Superintendent of Colindale Hospital, London) I analysed in 1940 a series of 428 cases of tuberculous laryngitis which I had observed at Colindale—the largest series of cases of this disease that has been published in Britain. We found then that 15·08 per cent. of the patients suffering from pulmonary tuberculosis had the larynx involved; this contrasts with the figure of 18·77 per cent. which Sir St. Clair Thomson had found in 1924—but contrasts even more with the figure of 2 per cent. given by Professor Ormerod in 1954. Why should the incidence of tuberculous laryngitis have dropped in such a striking fashion? It is not only because of the introduction of streptomycin and the other antibiotics, for the drop was evident before they ever appeared upon the scene. The main reason, I believe, is the general improvement in the social standards of the population of our country, for, although tuberculosis is caused by a bacillus, it is due even more due to a poor family history, malnutrition, bad housing, and poverty. Another reason is the more efficient treatment of the disease, mainly from the surgical aspect: one sanatorium which I used to visit near London had not, in 1932, even an operating theatre, while in 1953 no fewer than 75 per cent. of the patients received surgical treatment of one degree or another.

The antibiotics have changed the whole outlook in tuberculous laryngitis. The series of 428 cases of Professor Heaf and myself extended over the five years immediately prior to the Second World War; but when, five years after the war, I tried to analyse another series of cases I gave up the attempt, because there were so few cases and none of them were advanced. At the first sign of huskiness or loss of voice the medical officer was accustomed to order streptomycin and PAS.

### ADVANCES IN OTOLOGY

Only sixteen years ago, in January, 1939, the late Walter G. Spencer, a scholar and a historian as well as an eminent surgeon, wrote in the article on surgery in an anniversary number of *The Medical Press* the following words: "Otologists seem taken up with minutiae about the mastoids . . . it is a depressing specialty." Walter Spencer was of course, wrong.

Two years before, Professor Maurice Sourdille, then of Nantes, had demonstrated his fenestration operation, on which he had been working for some years, at the Summer Meeting at Norwich of the Section of Otology of the Royal Society of Medicine, though it was received with small enthusiasm until Julius Lempert of New York streamlined it during the next year. In 1935 Mr. Mollison was already curing Ménière's disease by injecting alcohol into the external semicircular canal of the labyrinth—a method that Terence Cawthorne has improved upon in recent years with marked success in some hundreds of cases; and Cairns and Hallpike published their revolutionary paper on the cause of Ménière's disease in 1938. Ballance and Duel had published their successful method of nerve-grafting for facial paralysis in 1932. It was so long before as 1926 that Rudolf Magnus of Utrecht had described in *The Lancet* his fundamental experimental work upon the labyrinth, and W. J. McNally of Montreal, C. S. Hallpike of London, and other otologists had been making notable contributions to this aspect of otology from 1925 onwards. So that Walter Spencer, scholar and historian though he was, had obviously not been reading the otological literature. Otology is not mere handicraft—it is a branch of philosophy.

### HEARING AIDS

Widely as the National Health Service has laid itself open to criticism—and I hesitate to think what Mr. Gladstone, that financial precisian, would have said about a Cabinet Minister who budgeted foı a scheme to cost £132,000,000 per annum and which in its last year under his direction cost £460,000,000—it has at least awakened ear, nose and throat specialists in general in this country to the importance of careful testing of the hearing under proper conditions and to the value of hearing-aids in many distressing cases of deafness, especially when supplemented by instruction in speech-reading and auditory training. In this country before July 1948 there were only four more or less adequately equipped deafness clinics, though another dozen did help to provide hearing-aids in one way or another, sometimes only by a fortnightly visit from a hearing-aid dealer. Now there are fifty hearing-aid or deafness centres, and by the end of 1954 over 400,000 deaf patients in England and Wales alone had been provided with a Government hearing-aid. I hold no brief for the National Health Service, but one must acknowledge what it has done not only for the deaf people of Britain but also in stimulating among otologists an interest in the rehabilitation of the deaf.

There have been great developments in hearing-aids in quite recent years, most of the improvements being due to those people who are somewhat unkindly designated the "commercial" hearing-aid makers. The first practical electric hearing-aid was made before my time as a doctor, in 1898, but I can remember the first portable aid, made by the Marconi Company in 1924, which weighed about 28 lb. The most recent improvement has been the development in the Bell Telephone Laboratories of the "transistor", the efficiency of which is now substantiated and which is already taking the place in hearing-aids of the small thermionic valve. It consists of a tiny piece of the rare metal germanium, suitably prepared and mounted; this enables the high-tension battery to be withdrawn and only a cheap low-tension battery is required, which lasts for as long as 300 hours. Dr. Stacy Guild, that very wise research worker in otology at Johns Hopkins Hospital, Baltimore, once remarked to me that if hearing-aids had been as efficient twenty years ago as they are today, the fenestration operation would never have been heard of!

AUDITORY TRAINING

I take the opportunity of emphasizing, as I have done before, that there is little use in merely handing a deaf person a hearing-aid, Government-supplied or otherwise, and a deafness clinic that does not have a member of its staff capable of carrying out auditory training is not doing its duty to the community and is, indeed, wasting Government money. My old hospital, the Metropolitan Ear, Nose and Throat Hospital in London, instituted in 1937 the first hearing-aid clinic connected with a hospital. It kept going, under difficulties, all through the war, and was reorganized and re-equipped, with a full-time staff, in 1945. A full range of the commercial hearing-aids on the " approved list " of the National Institute for the Deaf were made available for trial and sold by the makers at reduced prices to the hospital patients. The almoner made herself familiar with sources from which funds could be obtained to provide aids for deaf persons who could not afford to buy them and she was happy and proud to state, when the scheme ended in July, 1948, that no deaf person had had to go without a hearing-aid for financial reasons only.

But on examining the statistics of our hearing-aid clinic in 1946 and 1947 we found that in 50 per cent. and 33 per cent. respectively of the cases, the hearing-aids prescribed were being returned within a week or two, in spite of the trouble taken by our well-trained and enthusiastic staff, who were spending an average of over an hour on each patient. And when we read that in America at a Naval Veterans' Aural Centre, as a result of a rehabilitation programme lasting a few weeks, 94 per cent. of the deaf patients were wearing their hearing-aids with satisfaction at the end of a year, my hospital board thought it worth while to send me to study the subject on the spot. Early in 1948 I visited all the aural rehabilitation centres as well as a number of aural clinics, from New York, Philadelphia and Washington to New Orleans, St. Louis and Chicago, and I returned to London fully convinced of the value of auditory training, not only from the point of view of the deaf patient, but also from the point of view of economy in the provision of hearing-aids.

If a patient is properly adjusted physically and psychologically to a hearing-aid and trained to make the most of his residual hearing with the help of the aid, he will continue to use the aid sensibly and, indeed, indefinitely. But he must realize its limitations as well as its advantages, for no hearing-aid can ever be as good as normal hearing; and he must be reassured about the difficulties of the hearing-aid, persuaded of its value, and instructed in its mechanics and its upkeep. In addition, he should receive instruction in speech-reading (which may be described as " lip-reading *plus* ", i.e. the study of the whole features and gestures of the person talking, as well as the movements of the lips), and, if necessary, in speech-correction, for a harsh, unmodulated voice, such as some deaf persons develop, can be almost as great a handicap in communication as the deafness. In order to carry out this considerable programme, the instructor in auditory training should be a trained teacher of the deaf as well as a trained audiometrician. Few deafness clinics in Britain are fortunate enough yet to possess such a member of their staff, but there is no doubt that she—it is usually a she—is essential for its economic working from a long-term point of view.

Auditory training is a subject that has long interested me, but it must be emphasized that it is *not* a treatment of deafness. It improves understanding by helping the methods of communication, and the deafness itself is not affected. In my younger days I was assistant to Dr. George Cathcart, who introduced into this country many years ago the electrophonoide method of Zund-Burguet, which was in essentials a method of auditory training, three octaves of sounds within the speech range being forced upon the ears by this electrical instrument by means of vibrating earpieces. Zund-Burguet, a Frenchman, was not a doctor and his method of auditory training was not looked upon kindly by French otologists, for it is true that the method was empirical. But it was from Zund-Burguet that I learned about the early endeavours of Itard and the elder Urbantschitsch to carry out some form of auditory training. Jean Marie Gaspard Itard was a Parisian aural surgeon who published a good textbook on hearing and diseases of the ear in 1821 and deserves to be remembered for his pioneer work on the education and rehabilitation of the deaf. He showed that by systematic practice in listening the born-deaf child acquired an increased power of auditory perception; first, the child began to realize the difference between noises, then between vowels, and then between consonants. It was tedious work, but Itard considered it well worth while.

Victor Urbantschitsch in 1895 published the opinion that it was possible to teach partial perception of speech sounds to children who appeared to be totally deaf, and pointed out that tests of hearing made after auditory training showed that less than 3 per cent. of the pupils tested actually were in fact totally deaf. The ideas of Urbantschitsch were not accepted by other otologists in his native Vienna, and he was looked upon as rather a crank on the subject of the education of the deaf.

I have long been somewhat sceptical about the accepted methods of educating and training deaf children in this country and abroad. I know that it was so long ago as 1880 that at the International Congress of Teachers of the Deaf at Milan methods other than the oral system were thrown out of the window "amid scenes of great enthusiasm". But I have often wondered whether we can give deaf children any more help than they are receiving at present? I know that at Gallaudet College, Washington, D.C., the only educational institution for the deaf of university standard, they do not use the purely oral method, and I also know that there are a few sceptics among the teachers of the deaf in this country, though they make little headway against the "pure oralists" in authority. Then I read in 1951 an article by Wedenberg, who is a Swedish educationist, not an otologist, and he had come to the striking conclusion that it was important, in educating deaf children, to start auditory training before starting lip-reading; in fact, he thought lip-reading was a handicap towards education.

"In all cases described", he wrote, "'lip-reading' has already begun to play an important rôle for the children. It was, therefore, necessary to discourage it because, in the writer's opinion if the child grows too dependent upon speech-reading, he never learns to listen. The initial progress is slow with this procedure in comparison to that in the cases in which the patients, having learned to depend upon speech-reading from the beginning, achieved relatively rapid results. The handicap is gradually overtaken, however, by the 'auditory trained', who are considerably more auditorially focused and, therefore, more normal."

The idea of auditory training for young children is to enable the deaf child to develop hearing and speech in the same way as the normal child would do; that is to say, to listen first and then to imitate the sounds he is able to hear—for a sound is only learnt by constant repetition. In the first twelve months of life an infant gradually comes to understand some speech through continual listening, though does not himself speak yet; but this listening period is indispensable in the process of the child learning to speak. The age of about 12 to 18 months has been called the period of "readiness to speak", and understanding must always precede speaking.

A very severe degree of deafness need not prevent a child from learning, for 95 per cent. of so-called born-deaf children have some residual hearing: sometimes for low tones (i.e. the vowels), sometimes for high tones (i.e. the consonants). A very young child can be taught to use and appreciate a hearing-aid more easily than an older child—I know that Miss Whetnall, the otologist at the Royal National Hospital audiology centre, has successfully fitted a hearing-aid, of the small modern monopack type, to a child aged ten months.

Dr. George Cathcart's ideas on auditory education or re-education had failed to be generally accepted chiefly, I think, because he wrongly called it a treatment of deafness and because he devoted the method in the main to adults—I say, " in the main ", because on looking up the second edition of Hunter Tod's *Diseases of the Ear*, which Dr. Cathcart edited and indeed completely rewrote in 1925, I read that by the Zund-Burguet method of auditory re-education he " enabled three boys and one girl to be educated at ordinary schools; the various parents had been advised by several aurists to send them to a school for deaf-mutes." I may say that two of those boys, now middle-aged men, I number among my friends, for I got to know them well, long ago: one is head of an important business in London and the other is a solicitor in active practice in the provinces.

Why, then, did I not continue with the method advocated so strongly by George Cathcart? One reason was that George Cathcart himself continued to employ it regularly with deaf patients right up to the time of his death, at an advanced age, in 1951. Another reason was that it is a tedious and time-consuming method: deaf patients used to come for thirty daily treatments, twice a year if possible—even George Cathcart, for all his enthusiasm, used to hand over most of his patients to an assistant whom he trained. I read a paper on the subject at the B.M.A. Annual Meeting in 1930, but the whole idea was severely criticized by other otologists, I was strongly advised by friends among them not to be too closely associated with the method—which, coming from a non-medical source, was thought to have an element of quackery about it—and I, being a good deal younger then, thought it wiser not to send in the paper for publication.

In quite recent years, with the development of hearing-aids and their widespread use, there has been an increased interest in auditory training, but the emphasis has switched from the adult to the very young. Deaf children have been systematically taught lip-reading for very many years—it began with a Spanish Benedictine monk in the sixteenth century, and the first schools for deaf children were established in France, Germany and Scotland in the eighteenth century—and the age at which it is begun has gradually become earlier, intil the Education Act of 1944 has made pre-school education of deaf children permissive from the age of two.

An important milestone was the realization that a born-deaf child, like a normal child, should get his earliest education from his mother, as early as the second six months of life, when the normal child is beginning to babble and to educate himself from the sounds he hears around him. The importance of training the parents of a deaf child to educate the child was first realized, not by some eminent otologist or educator of the deaf, but a mother, Louise Treadwell Tracey. She is not only the wife of one of the greatest actors among film stars, Spencer Tracey, but also the mother of a son, now in his twenties, who was born deaf. From her own personal problem she evolved the important principle that not only must the education of a deaf child begin at the earliest possible moment, but that the parents of the born-deaf child must be educated as well as the child himself.

Along with two other mothers of deaf children, in 1942 Mrs Tracey started a clinic in a small house at Los Angeles—it was not a regular school, for the children were of pre-school age. She soon found, however, that the training given at her clinic was often defeated when the child returned home, and this led her to experimenting with the education of children and parents simultaneously. The clinic was not a centre where parents learned to become teachers or to take their place, but was intended to encourage and aid them and to assist with home training and supplementary work. Success proved immediate, and even more widely successful has been the fascinating correspondence course for parents which Mrs. Tracey planned, and which has now been used by some 4,500 parents of deaf children all over the world.

The parents of deaf children get out of auditory training largely what they bring to it, and it takes a great deal of patience and time and devotion. When the fact of deafness is first discovered there is usually a tendency on the part of the parents to stop talking to the child. That is wrong. The whole family should go on talking to him just as if he could hear, but they must not expect any answer or even any indication that he understands. In time—and it will seem a long time—the child will actually begin to understand much of what is being said to him, even though he does not hear it, and gradually he will begin to respond. There is no use being in a hurry for the deaf child to learn, it takes time to explain things to him. Repetition, repetition, and again repetition is the only secret of teaching language to a deaf child.

### CONCLUSION

What, then, of the future? It has been suggested in some quarters that the specialty of oto-laryngology is on the decline: that the audiologist with his hearing-aids is usurping the functions of the otologist, the allergist is taking over the nose and nasal sinuses, the neuro-surgeon should do the surgery of brain abscess, the radiotherapist is making the surgery of the larynx obsolete, bronchoscopy has slipped from the hands of the laryngologist to those of the thoracic surgeon and oesophagoscopy to the hands of the gastro-enterologist, and most of all, that penicillin and the newer antibiotics have taken the bread out of honest ear, nose and throat surgeons' mouths. In spite, however, of the baleful influence of the dead hand of officialdom on medicine in this country, I am optimistic about the future of oto-laryngology. Only a few days ago I saw the large Barnes Hall of the Royal Society of Medicine in London crowded to the doors by eager-looking young men and women, for the meetings of the Sections of Otology and of Laryngology. And I contrast this with my memory of the first meeting at which I spoke in the Section of Otology in June, 1925—it was on the subject, curiously enough, of hearing-aids, with Dr. Kerr Love of Glasgow in the chair—when some thirty otologists were present in the more than adequate smaller hall.

The otologist used habitually to be a pessimist. I remember my old teacher, Dr. Logan Turner of Edinburgh, saying to me: "Wait until you are my age, and you'll be a pesssimist too." Well, now I am his age and oto-laryngology is more alive today than ever. It is more alive than ever because it is more hopeful than ever, and its practitioners look forward optimistically to the solution of more and more of its fascinating and stimulating problems.

### REFERENCES

Andrews, A. (1953). In *The Education of Deaf Children*, pp. 2–5, London.

Ashcroft, D. W., and Hallpike, C. S. (1934). *Journ. Laryng. and Otol.*, **49**, 450.

Cathcart, G. C. (1925). *Lancet*, **1**, 968.

Cathcart, G. C. (1926). *Hunter Tod's Diseases of the Ear*, 2nd edition, p. 310, London.

Colledge, L. (1945). *Journ. Laryng. and Otol.*, **60**, 419.

Fleming, A. (1929). *British Journ. Exper. Path.*, **10**, 226.

Florey, H. W., Chain, E., *et al.* (1940). *Lancet*, **2**, 226.

Fry, D. B., and Whetnall, E. (1954). *Lancet*, **1**, 583.

Itard, J. M. G. (1821). *Traité des maladies de l'oreille et de l'audition*, Paris.

Lederman, M. (1954). *Journ. Laryng. and Otol.* **68**, 333.

Magnus, R. (1926). *Lancet*, **2**, 551, 585.

Ormerod, F. C. (1954). Semon Lecture, *Journ. Laryng. and Otol.*, **58**, 1.

Ormerod, F. C. (1954). In Ellis's *Modern Trends in Diseases of the Ear, Nose and Throat*, p. 316, London.
Spencer, W. G. (1939). *Medical Press*, **201**, 100.
Stevenson, R. Scott (1935). *Recent Advances in Laryngology and Otology*, London.
Stevenson, R. Scott (1946). *Morell Mackenzie*, p. 175. London.
Stevenson, R. Scott (1949). *Recent Advances in Oto-laryngology*, 2nd edition, London.
Stevenson, R. Scott (1953). *Teacher of the Deaf*, **51**, 34.
Stevenson, R. Scott, and Heaf, F. R. G. (1940). *Brit Med. J.* **1**, 164.
Urbantschitsch, V. (1895). *Über Horübungen bei Taubstummheit*, Vienna.
Watson-Williams, P. (1894). *Diseases of the Upper Respiratory Tract*, Bristol.
Watson-Williams, P. (1930). *Chronic Nasal Sinusitis and its relation to General Medicine* (2nd edition, 1933), Bristol.
Yates, A. Lowndes (1924). *Journ. Laryng. and Otol.*, **39**, 554.

